# Radioembolization with Y-90 Glass Microspheres: Do We Really Need SPECT-CT to Identify Extrahepatic Shunts?

**DOI:** 10.1371/journal.pone.0137587

**Published:** 2015-09-03

**Authors:** Jens M. Theysohn, Marcus Ruhlmann, Stefan Müller, Alexander Dechene, Jan Best, Johannes Haubold, Lale Umutlu, Guido Gerken, Andreas Bockisch, Thomas C. Lauenstein

**Affiliations:** 1 Department of Diagnostic and Interventional Radiology and Neuroradiology, University Hospital Essen, Essen, Germany; 2 Department of Nuclear Medicine, University Hospital Essen, Essen, Germany; 3 Department of Gastroenterology and Hepatology, University Hospital Essen, Essen, Germany; The University of Chicago, UNITED STATES

## Abstract

**Purpose:**

Selective Internal Radiation Therapy (SIRT) with ^90^yttrium (Y-90) is an increasingly used therapeutic option for unresectable liver malignancies. Nontarget embolization of extrahepatic tissue secondary to vascular shunting can lead to SIRT associated complications. Our aim was to assess whether extrahepatic shunts can reliably be diagnosed based on hepatic digital subtraction angiography (DSA) or whether subsequent SPECT/CT data can provide additional information.

**Materials and Methods:**

825 patients with hepatocellular carcinoma (n = 636), hepatic metastases (n = 158) or cholangiocellular carcinoma (n = 31) were retrospectively analyzed. During hepatic DSA 128 arteries causing shunt flow to gastrointestinal tissue were coilembolized (right gastric artery n = 63, gastroduodenal artery n = 29; branches to duodenum / pancreas n = 36). Technectium-99m-labeled human serum albumin (HSA) was injected in all 825 patients. SPECT/CT data was used to identify additional or remaining shunts to extrahepatic tissue.

**Results:**

An unexpected uptake of HSA in extrahepatic tissue was found by SPECT/CT in 54/825 (6.5%) patients (located in stomach n = 13, duodenum n = 26, distal bowel segments n = 12, kidney n = 1, diaphragm n = 2). These patients underwent repeated DSA and newly identified shunt vessels were coilembolized in 22/54 patients, while in 12/54 patients a more distal catheter position for repeat injection of HSA was chosen. In 20/54 patients the repeated SPECT/CT data still revealed an extrahepatic HSA uptake. These patients did not receive SIRT.

**Conclusion:**

Most extrahepatic shunts can be identified on DSA prior to Y-90 therapy. However, SPECT-CT data helps to identify additional shunts that were initially not seen on DSA.

## Introduction

Selective Internal Radiation Therapy (SIRT), also referred to as radioembolization (RE), is an increasingly used therapy for advanced primary and secondary liver malignancies [1,2,3]. Microspheres labeled with the beta-emitter yttrium-90 (Y-90) are injected into liver arteries using a catheter technique. The microspheres are trapped within the microvasculature of the tumor. Thus, particularly hypervascularized tumors can be treated with tumoricidal radiation doses while sparing non-neoplastic liver tissue [4,5].

The incidence of side-effects associated with SIRT is relatively low [6]. It may include vascular injuries during DSA, hepatobiliary toxicity / liver failure and postradioembolization syndrome (fatigue, nausea, vomiting) [7,8,9,10]. Furthermore, complications can occur when microspheres are deposited in extrahepatic tissue. The latter may lead to severe organ damage, particularly of the gastrointestinal tract or the pancreas. It is therefore mandatory to identify shunt vessels arising from the hepatic arterial tree supplying extrahepatic organs. These vessels should be either occluded by coil embolization prior to SIRT or a safe infusion side of the microspheres distal to the origin of the shunt vessel has to be chosen [11,12,13].

Prior to the actual Y-90 administration a test run is performed using technetium-99m-labeled human serum albumin (99mTc-HSA) injected intraarterially into the liver followed by SPECT, SPECT/CT or planar imaging. The rationale for this step is threefold: first, hypervascularity of hepatic tumor lesions is documented. Second, shunting to the lung can be quantified, which must not exceed a certain dose [14,15]. Third, 99mTc-HSA imaging can be used to visualize extrahepatic abdominal tracer deposition if shunt vessels have not been identified during DSA. In such cases hepatic angiography is being repeated in order to change catheter positions or coil-embolize additional vessels until no extrahepatic 99mTc-HSA deposition occurs [11,16].

Currently, there is some debate about the value of 99mTc-HSA studies prior to SIRT. Several aspects can be discussed: (a) according to the literature, there is a wide range in the incidence of extrahepatic albumin accumulation prior to SIRT, reported to be between single-digit numbers and 42% [17,18,19] with SPECT/CT being a more sensitive tool than SPECT or planar imaging [20]. (b) In the study reporting the highest number of patient (n = 341; [16]), however, only SPECT was used and the incidence of extrahepatic albumin accumulation was as low as 9.7%. (c) There are concerns that extrahepatic 99mTc-HSA deposition has no clinical value at all if no collaterals were detected by means of DSA [21]. Hence, the aim of this study was to assess the incidence of extrahepatic 99mTc-HSA deposition utilizing SPECT/CT and the need to repeat DSA prior to SIRT in a large patient cohort.

## Materials and Methods

The University Hospital Essen ethics committee approved the retrospective, anonymous analysis of this data. The study has been conducted according to the principles expressed in the Declaration of Helsinki.

### Patient population

Between 2007 and 2014 825 patients (611 male, 214 female, mean age 65.9 years) were scheduled for SIRT using TheraSphere™ (BTG, London, UK). Patients suffered from tumor entities including hepatocellular carcinoma (HCC; n = 636), hepatic metastases (n = 158) and cholangiocellular carcinoma (CCC; n = 31). All patients were not amenable to curative treatments due to advanced tumor disease or other medical reasons. Informed consent for angiography and SIRT was obtained.

### Pretreatment angiography

A biplane digital subtraction angiography (DSA) system (Toshiba Infinix DP-i, Toshiba Medical Systems, Tokio, Japan or Philips Allura™, Philips Healthcare, Best, Netherlands) was used for the pretreatment angiography of the hepatic arteries. First, a 5-French guiding catheter (Sidewinder-1, Sidewinder-2 or Cobra-2, Terumo Europe, Leuven, Belgium) was inserted applying a Seldinger technique via a transfemoral access. Second, selective angiographies of the celiac trunk and the superior mesenteric artery were performed. Intraarterial contrast (Ultravist 300, Bayer Healthcare, Berlin, Germany or Xenetix 300, Guerbet, Roissy, France) was administered in a dose of 15cc and an injection rate of 5cc / s utilizing an automatic injector (Tyco Healthcare, Mansfield, MA, USA). Imaging was performed with 80 kV and 70 mAs, the acquisition rate was 2 images per second. If detectable, extrahepatic shunting vessels were either occluded by permanent coilembolization or a more distal catheter position for the injection was chosen. For coilembolization a 3-French microcatheter (Renegade, Boston Scientific, Natick, MA, USA) was used and the shunt vessel was occluded with microcoils (IDC coils, Boston Natick, MA, USA). Complete coilembolization was documented with subsequent contrast series.

After identifying the extent of tumor load, the catheter position(s) for the intended treatment were chosen including the right and/or left liver lobe. A micro catheter (Rebar 0.27 inch; ev3 Plymouth, MN, USA) was used for this purpose. A total of 150 MBq ^99m^Tc-Human serum albumin (^99m^Tc-HSA; ROTOP Pharmaka AG, Radeberg, Germany) was injected from this or these position(s).

### SPECT/CT

Patients were referred to the Department of Nuclear Medicine after the administration of ^99m^Tc-HSA. SPECT/CT images were acquired on a Symbia™ SPECT/CT system (Siemens Healthcare, Erlangen, Germany) on average 60 minutes after the injection of ^99m^Tc-HSA. Imaging parameters were as follows: matrix of 128x128 with 128 frames (25s/frame), CT: 128 kV, 17 mAs, slice thickness: 5 mm, image reconstruction with a medium smooth kernel. SPECT images were visualized in 3 orthogonal planes and were corrected for attenuation and scatter. Fusion images were created from the co-registered SPECT and low-dose CT images using the e.soft™ 2007 application package (Siemens Healthcare, Erlangen, Germany). Extrahepatic tracer accumulation was evaluated by specialists in nuclear medicine and board certified radiologists in consensus during the following weekly tumor board at our institution.

### Repeated angiography and SPECT/CT

In patients with extrahepatic ^99m^Tc-HSA uptake, a repeat DSA was performed in the same manner as described above. Based on the initial SPECT/CT as well as angiographic findings, shunt vessels were to be identified and coilembolized if possible. Alternatively, the microcatheter was placed in a more distal position for the repeated ^99m^Tc-HSA injection thereby avoiding the injection of microspheres in more proximal shunt vessels. As for the initial angiography, SPECT/CT was performed following the repeated ^99m^Tc-HSA injection.

### Study data analysis

In a first step, the number of patients with unexpected extrahepatic tracer deposition was calculated and the according anatomical region of tracer deposition was noted (gall bladder and abdominal wall (falciform artery) were not included into our analysis). Subsequently, the number of shunt vessels that was depicted in the repeated angiography was calculated and consequences for the therapeutic concept (coilembolization / change in catheter position / withdrawal from SIRT) were determined.

## Results

During pretreatment angiography 128 potential shunt vessels to extrahepatic tissue in 112 out of 825 patients were coilembolized. These vessels encompassed the right gastric artery (n = 63), the gastroduodenal artery (n = 29) or small shunt branches to the small bowel (n = 36).

An unexpected increased 99mTc-HSA uptake in extrahepatic tissue was found in 54/825 (6.5%) patients (stomach n = 13, duodenum n = 26, distal bowel segments n = 12, kidney n = 1, diaphragm n = 2). These patients underwent repeat DSA after a mean 11 days following the initial DSA. Shunt vessels were identified in 34/54 patients. These vessels were coilembolized during the repeated DSA in 22/54 patients, while in 12/54 patients a more distal catheter position was chosen. In the remaining 20/54 patients the repeated SPECT/CT data still revealed an extrahepatic 99mTc-HSA uptake, although coilembolization had been performed in 15 and the catheter position had been changed in 5. These patients were excluded from SIRT (20/825, 2.4%).

Furthermore, 117 patients were excluded from SIRT due to insufficient hyperperfusion of the tumors (n = 32) or clinical reasons (n = 85). Hence, the overall completion rate of SIRT amounted to 83.4% (688 out of 825).

**Fig 1:**
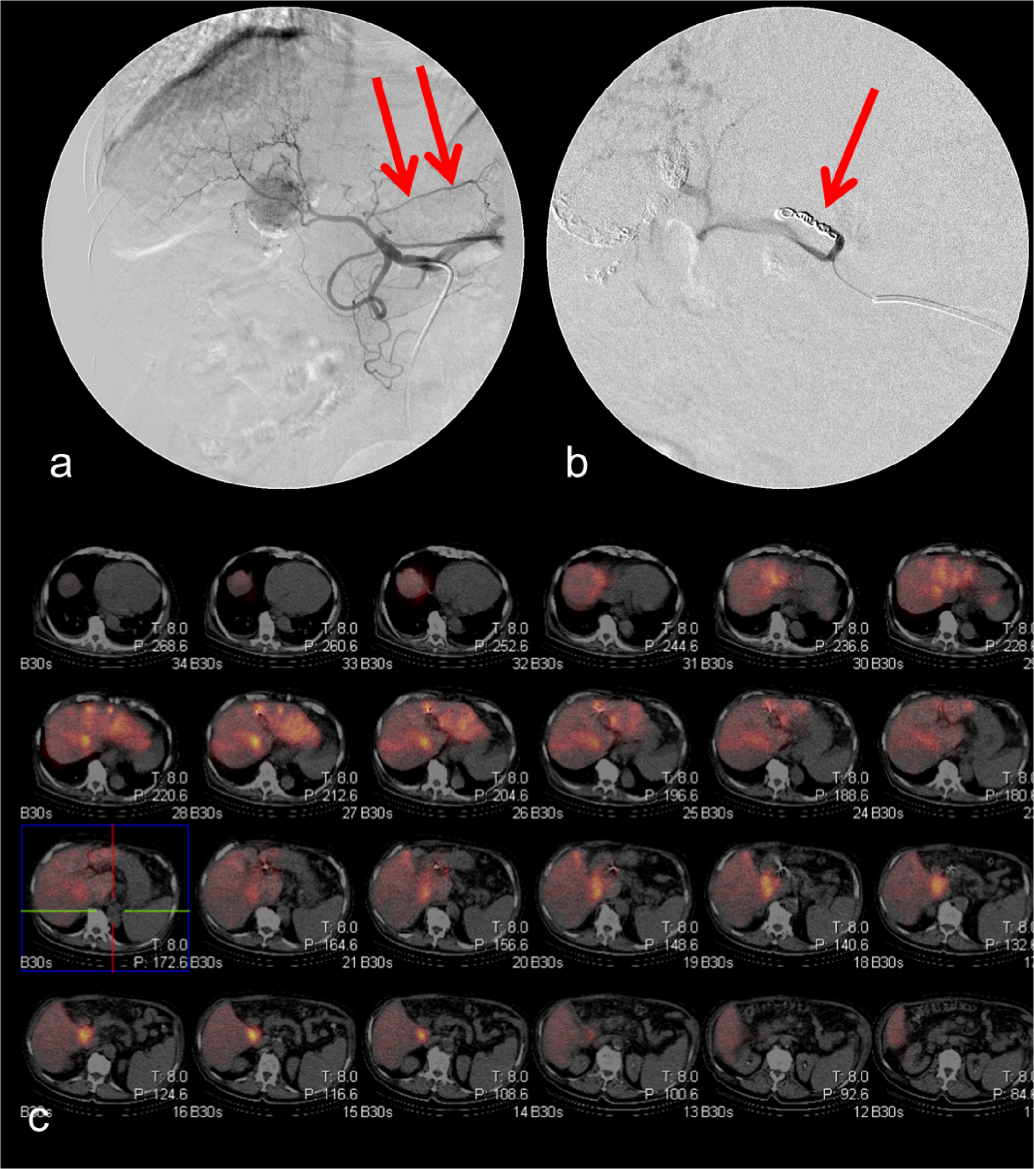
65 year-old male patient. The right gastric artery (Fig 1a; arrow) was identified and coilembolized (Fig 1b). SPECT/CT (Fig 1c) did not reveal extrahepatic tracer deposition.

**Fig 2:**
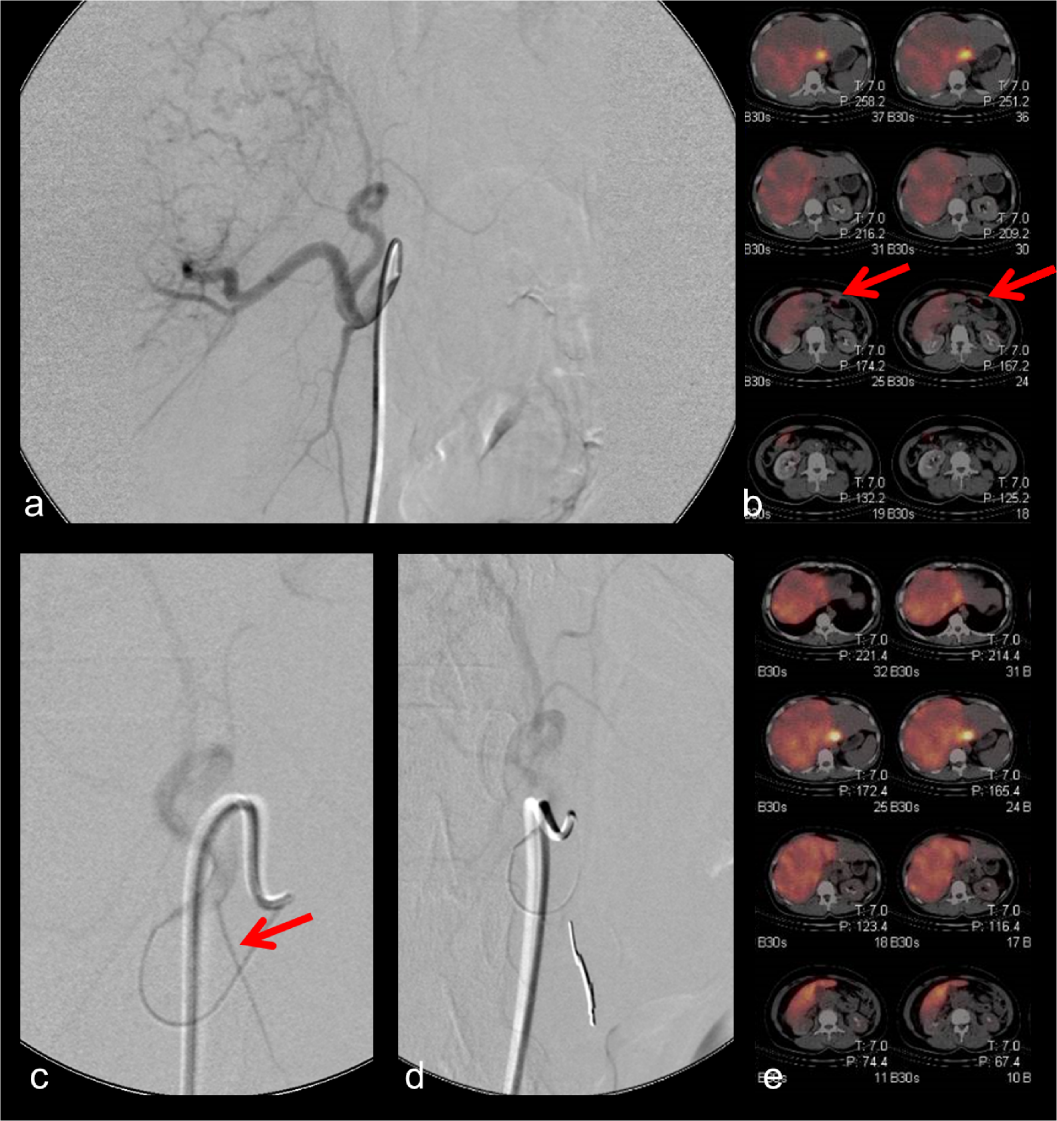
41 year-old female patient. Initial DSA did not reveal any shunt vessels (Fig 2a). However, 99mTc-HSA scan revealed extrahepatic tracer uptake at the gastroduodenal junction (Fig 2b). A shunt vessel was identified during repeated DSA and occluded (Fig 2c) and no extrahepatic 99mTc-HSA deposition was seen anymore (Fig 2d).

**Fig 3:**
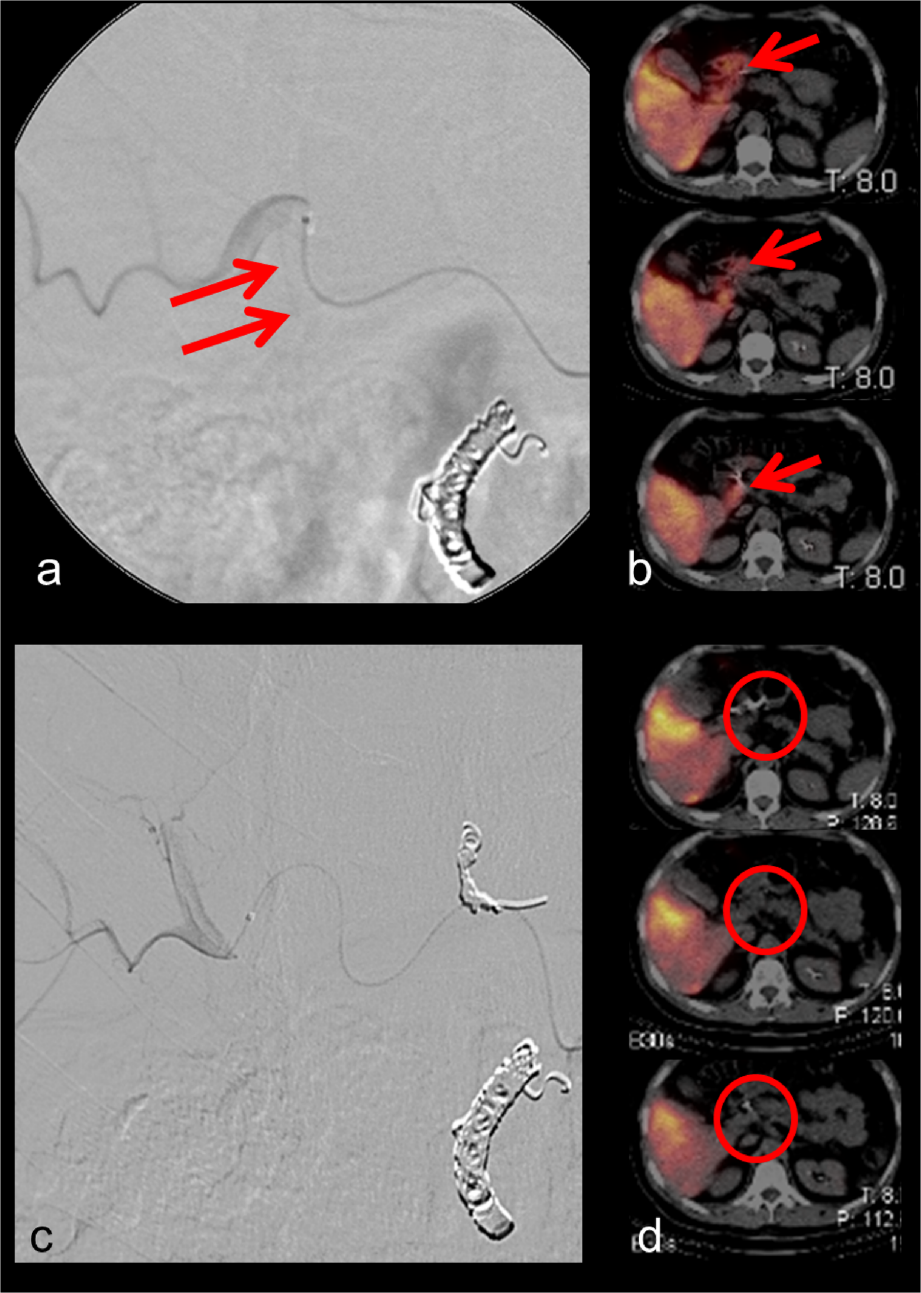
47 year old male patient. During the initial DSA the gastroduodenal artery and right gastric artery were occluded. However, a small branch to the small bowel arising from the right hepatic artery was missed (Fig 3a) resulting in an extrahepatic 99mTc-HSA deposition in the small bowel wall (Fig 3b). During repeated angiography the microcatheter was positioned in a more distal part of the right hepatic artery (Fig 3c). Subsequent SPECT/CT did not reveal any extrahepatic tracer accumulation (Fig 3d).

Figs 1–3 display different examples of our patient cohort, each with hepatocellular carcinoma. In Fig 1 the case of a 65 year old patient is shown. During pretreatment angiography the right gastric artery was coilembolized. No extrahepatic 99mTc-HSA uptake was seen in the corresponding SPECT/CT scans. Hence, no repeated angiography was needed. Fig 2 provides data of a 41 year old patient. During the initial DSA no shunt vessels were coiled. 99mTc-HSA scan revealed extrahepatic tracer uptake at the gastroduodenal junction. During the repeated angiography a shunt vessel arising from the left hepatic artery was identified and occluded. The subsequent second SPECT/CT did not show any extrahepatic 99mTc-HSA deposition. Fig 3 represents the case of a 47 year old patient. During the initial DSA the gastroduodenal artery and right gastric artery were occluded. However, a small branch to the small bowel arising from the right hepatic artery was missed. Hence, SPECT/CT exhibited extrahepatic 99mTc-HSA deposition in the small bowel wall. During repeated angiography the microcatheter was positioned in a more distal part of the right hepatic artery thereby avoiding the perfusion of the shunt vessel with microspheres.

## Discussion

The current study carries two messages we believe to be important. First, SPECT/CT prior to SIRT helped to identify additional extrahepatic shunts vessels in 6.5% of the examinations. Data of our study is based on a number of patients that was considerably higher than in previous trials. Secondly, therapeutic strategies may subsequently be adapted by coilembolizing these shunt vessels or by changing the infusion site of microspheres. Eventually, only a small minority of patients must be excluded from SIRT for technical reasons.

Minor side-effects following SIRT include fatigue, pain, nausea or fever [9]. The incidence of severe complications, such as gastrointestinal ulcerations due to extrahepatic microsphere deposition, is reported to be relatively low [22,23]. Peterson et al. described in a series of 112 procedures the incidence of gastrointestinal ulcerations to be as high as 10% including grade 3 or even grade 4 complications [9]. Other authors report on a lower number of gastroduodenal ulcers ranging between 1 and 3% of all procedures [8,10,24]. Despite the low overall incidence, impact of extrahepatic microsphere deposition on patients’ quality of life is considerable. Hence, all measures should be taken to prevent such complications associated with Y-90 therapy.

There is consensus that SPECT/CT following 99mTc-HSA administration offers a very high sensitivity and specificity for the depiction of extrahepatic tracer accumulation with significant advantages compared to planar imaging or SPECT alone [20]. The disadvantage of additional radiation does exposure through the low-dose CT part accounts for roughly 1 millisievert [mSv] and is not considered crucial in tumor patients. Hamami et al. compared SPECT/CT with SPECT and planar imaging in a series of 68 SIRT procedures [18]. Gastrointestinal shunting was revealed in 4 patients by planar imaging, in 9 patients by SPECT and in 16 patients by SPECT/CT. A comparable trial was performed by Ahmadzadehfar et al. who analyzed 90 hepatic angiograms prior to SIRT [19]. Extrahepatic tracer accumulation was found by planar imaging in 12%, by SPECT in 17% and even in 42% by SPECT/CT. The high incidence of extrahepatic shunting detected by SPECT/CT may seem surprising when compared to the results described in our study. One explanation could be related to the fact that data of Hamami et al. and Ahmadzadehfar et al. were collected several years ago when experience with SIRT was to still limited.

Our results are mostly in keeping with data published by Jiang et al. [17]. They examined a smaller cohort of 159 patients. Abnormal extrahepatic Tc-99m HSA distribution in extrahepatic organs was detected by SPECT/CT in 11%. In most patients the cause of abnormal deposition was identified by reviewing the pretreatment DSA. In a trial reporting on 341 patients SPECT was co-registered with MRI [16]. In 33 patients (9.7%) extrahepatic tracer deposition was found. Interestingly, the authors also describe the consequences of these findings. At repeat DSA the source of extrahepatic flow was detected in 31 patients. Consecutively shunts were coilembolized in 20 patients and/or a more distal microcatheter position was used (13 patients). Finally, extrahepatic 99mTc-HSA deposition was eliminated in 30 out of 33 patients. Although there are several parallels to our study and consequences drawn from the extrahepatic tracer accumulation are similar, data is only partially comparable to our findings based on SPECT/CT.

Gates et al. describe a new strategy where shunt vessels are mainly identified based on DSA and planar imaging is only used for the calculation of lung shunt fraction [21]. There is one very appealing aspect of this new approach: only one hospital stay has to be planned as the pretreatment DSA and treatment with Y-90 can be realized on a single day. A pilot study encompassed 14 patients proving the feasibility of this concept. There were no reportable side-effects. However, there are some aspects that can be controversially discussed. Examinations were performed in a center with a very high experience in SIRT. Hence, results cannot fully be transferred to any other radiology side. Furthermore, only a comparatively low number of patients were examined and this concept has to be evaluated in a larger patient cohort.

Still, there is debate whether SPECT/CT is necessary to identify clinically relevant shunt vessels. It is conceivable that very small shunt vessels lead to a negligible amount of extrahepatic microsphere deposition resulting in no or subclinical symptoms. However, it is very difficult to predict whether a shunt vessel would be clinically relevant. Furthermore, there may be differences between the use of glass and resin microspheres as the latter have a more pronounced embolic character due to a 50 times smaller dose per microsphere and the need for a larger amount of these to achieve the treatment dose. It may lead to stasis or even re-flow, which in itself is a risk factor for extrahepatic tracer accumulation [24]. As newer imaging techniques as C-arm angiography bring about a higher detection rate of smaller shunt vessels during DSA [25,26], future studies comparing this to SPECT/CT data could be interesting.

Clearly, our study is not without some limitations. We did not use C-arm angiography, which has been shown to accurately detect additional shunt vessels during DSA [25,26]. When applying C-arm angiography, one can assume that the incidence of extrahepatic tracer deposition will be lower than 6.5% as described in our study since more shunt vessels are identified and occluded during pre-treatment DSA. Furthermore, we only considered gastrointestinal shunt vessels, while the cystic artery and falciform artery, which may also represent extrahepatic shunt vessels, were excluded from our analysis. However, perfusion of either cystic or falciform artery has only minor clinical importance as possible side effects such as microsphere deposition in the abdominal wall or gallbladder wall will usually not lead to severe complications [27,28,29,30]. Finally, all extrahepatic HSA accumulations were judged as real and made generation and evaluation of false positive cases impossible, since patients with extrahepatic HSA deposition were never treated with SIRT.

In conclusion, most shunt vessels prior to SIRT can be identified on DSA and measures can be taken to prevent extrahepatic microsphere deposition. Yet, SPECT/CT is very helpful to identify additional gastrointestinal shunt vessels that were missed on DSA. A repeat DSA should be performed in these cases and most patients are subsequently eligible for SIRT.
